# Smartphones enabled up to 58 s strong-shaking warning in the M7.8 Türkiye earthquake

**DOI:** 10.1038/s41598-024-55279-z

**Published:** 2024-02-28

**Authors:** Francesco Finazzi, Rémy Bossu, Fabrice Cotton

**Affiliations:** 1 Department of Economics, University of Bergamo, Bergamo, Italy; 2 European-Mediterranean Seismological Centre, Arpajon, France; 3 CEA, DAM, DIF, 91297 Arpajon, France; 4 GFZ German Research Centre for Geosciences, Potsdam, Germany; 5 Institute of Geosciences, University of Potsdam, Potsdam, Germany

**Keywords:** Natural hazards, Seismology

## Abstract

Public earthquake early warning systems (PEEWSs) have the potential to save lives by warning people of incoming seismic waves up to tens of seconds in advance. Given the scale and geographical extent of their impact, this potential is greatest for destructive earthquakes, such as the M7.8 Pazarcik (Türkiye) event of 6 February 2023, which killed almost 60,000 people. However, warning people of imminent strong shaking is particularly difficult for large-magnitude earthquakes because the warning must be given before the earthquake has reached its final size. Here, we show that the Earthquake Network (EQN), the first operational smartphone-based PEEWS and apparently the only one operating during this earthquake, issued a cross-border alert within 12 s of the beginning of the rupture. A comparison with accelerometer and macroseismic data reveals that, owing to the EQN alerting strategy, Turkish and Syrian EQN users exposed to intensity IX and above benefitted from a warning time of up to 58 s before the onset of strong ground shaking. If the alert had been extended to the entire population, approximately 2.7 million Turkish and Syrian people exposed to a life-threatening earthquake would have received a warning ranging from 30 to 66 s in advance.

## Introduction

Public earthquake early warning systems (PEEWSs) combine seismic monitoring technologies and communication infrastructures to alert people of an imminent earthquake. PEEWSs are operational in only a few countries that are located in seismic zones^1^; their widespread use is limited by large implementation costs^2^ and legislative issues^3^. Smartphone-based PEEWSs are low-cost and crowdsourced^4^ alternatives to classic systems. The Earthquake Network (EQN)^5^ and the Android Earthquake Alerts System by Google^6^ are the two public smartphone-based PEEWSs operational at the global level. EQN became operational in 2013, with earthquake detection data routinely collected and stored from 2017 onwards^7^. Google’s system was initially deployed only in certain countries from 2021 onwards^8^.

The EQN alerting service is an opt-in one. Citizens must install the EQN app on their smartphones (Android, iOS or Huawei) to join the seismic monitoring and to receive alerts. When the smartphone is charging, the app enables seismic monitoring and data transfer to the EQN server infrastructure. During monitoring, the app analyses the smartphone's acceleration in real time to detect significant shaking relative to the sensor's background noise. Once a shaking starts, the app calculates the peak resultant acceleration over a 3-s window, hereafter called the smartphone peak resultant acceleration, and sends this information to the EQN server along with the smartphone coordinates. No waveforms are transmitted to the server. Monitoring by the app ends when the shaking is no longer distinguishable from sensor noise or when someone interacts with the smartphone.

When an earthquake is detected by the EQN system, smartphone peak resultant accelerations sent to the server are analysed to estimate the EQN median acceleration, infer the magnitude of the detected earthquake from this median acceleration and determine warning distances for mild, moderate, and strong shaking. EQN app users then receive an alert with a personalised countdown (with respect to the theoretical arrival of the S-wave) and expected ground-shaking intensity based on their location.

The EQN performance has been studied in terms of earthquake detectability^9^, alerting performance^7^ and user appreciation^10^. However, the effectiveness of smartphone-based PEEWSs has never been assessed during a large-magnitude (M > 7.5) destructive event in an inhabited area.

The magnitude 7.8 Pazarcik earthquake of 6 February 2023 was the fifth deadliest event in the last 20 years^11,12^. Türkiye does not have a national PEEWS but is covered by both the EQN and the Google systems. According to an investigation by the BBC (https://www.bbc.com/news/technology-66316462), the Google system failed to alert people. In contrast, the EQN system detected the earthquake and issued an alert to its users and on social networks (https://twitter.com/SismoDetector/status/1622404158618730498). The Pazarcik earthquake is the second largest destructive event—after the M 9.0 earthquake in Tohoku (Japan) in 2011—in terms of magnitude for which a public early warning was issued^13^ and also the first such onshore earthquake of this magnitude detected by a smartphone-based PEEWS for which data are available.

Large-magnitude earthquakes are challenging because of their long rupture length and duration. The rupture of the Pazarcik earthquake occurred over a fault length of 350 km and the total rupture duration was close to 80 s^14,15^. Most PEEWSs work by estimating shaking levels from rapidly determined earthquake parameters and issuing an alert to potentially affected people. Their performance is limited by how quickly the earthquake parameters and the uncertainty associated with the shaking level can be estimated, particularly for high intensity ground motions^16–18^. The EQN alerting strategy is similar to the onsite approach^19^, as it estimates alerting distances for different shaking levels from the first few seconds of the seismic waves without waiting for the end of the rupture.

This paper analyses and discusses the EQN alerting strategy and its warning performance during the Pazarcik earthquake. First, we present a detailed description of the operation of the EQN system during this major event (number of users at the time of the earthquake, number and location of detections, and information and timing of information sent to users). Second, the waveforms collected by the accelerometric stations of the Disaster and Emergency Management Authority (AFAD) of Türkiye are used to estimate the actual warning times offered by EQN to its Turkish and Syrian users with respect to a given ground shaking level. Third, we evaluate the number of Turkish and Syrian people who could have benefitted from these warning times if the alert had been issued to the entire population within the area where strong shaking was expected.

### Deployment and use of EQN before the Pazarcik earthquake

Before the Pazarcik earthquake, the EQN app had been downloaded more than 12 million times worldwide, and the number of active users is about 1 million worldwide and 33,000 in Türkiye. The EQN system has already issued 60 alerts worldwide for earthquakes of magnitude 5.5 and above. None of these earthquakes had an epicentre in Türkiye. However, EQN users located to the southwest of Türkiye received alerts for offshore earthquakes in Crete (Greece). These included a 5.7 magnitude earthquake on 29 December 2021 and a 5.5 magnitude earthquake on 20 November 2022.

### Pazarcik earthquake detection by the EQN

At the time of the Pazarcik event, 53 smartphones located within 100 km of the epicentre were monitoring for earthquakes. The EQN detection occurred at 01:17:46 UTC on 6 February 2023 with a delay of 12.1 s with respect to the origin time t0 given by U.S. Geological Survey (USGS). This was the time it took for the seismic waves to reach enough monitoring smartphones and for the EQN detection algorithm to recognise the ongoing earthquake.

The detection and assessment of the level of shaking was based on 20 of the 53 smartphones, mainly located in the cities of Gaziantep and Kahramanmaraş, approximately 37 km and 39 km from the epicentre. The EQN estimated epicentre (37.481° latitude, 36.997° longitude) had an error of 16.7 km with respect to the epicentre provided by the USGS.

**Figure 1:**
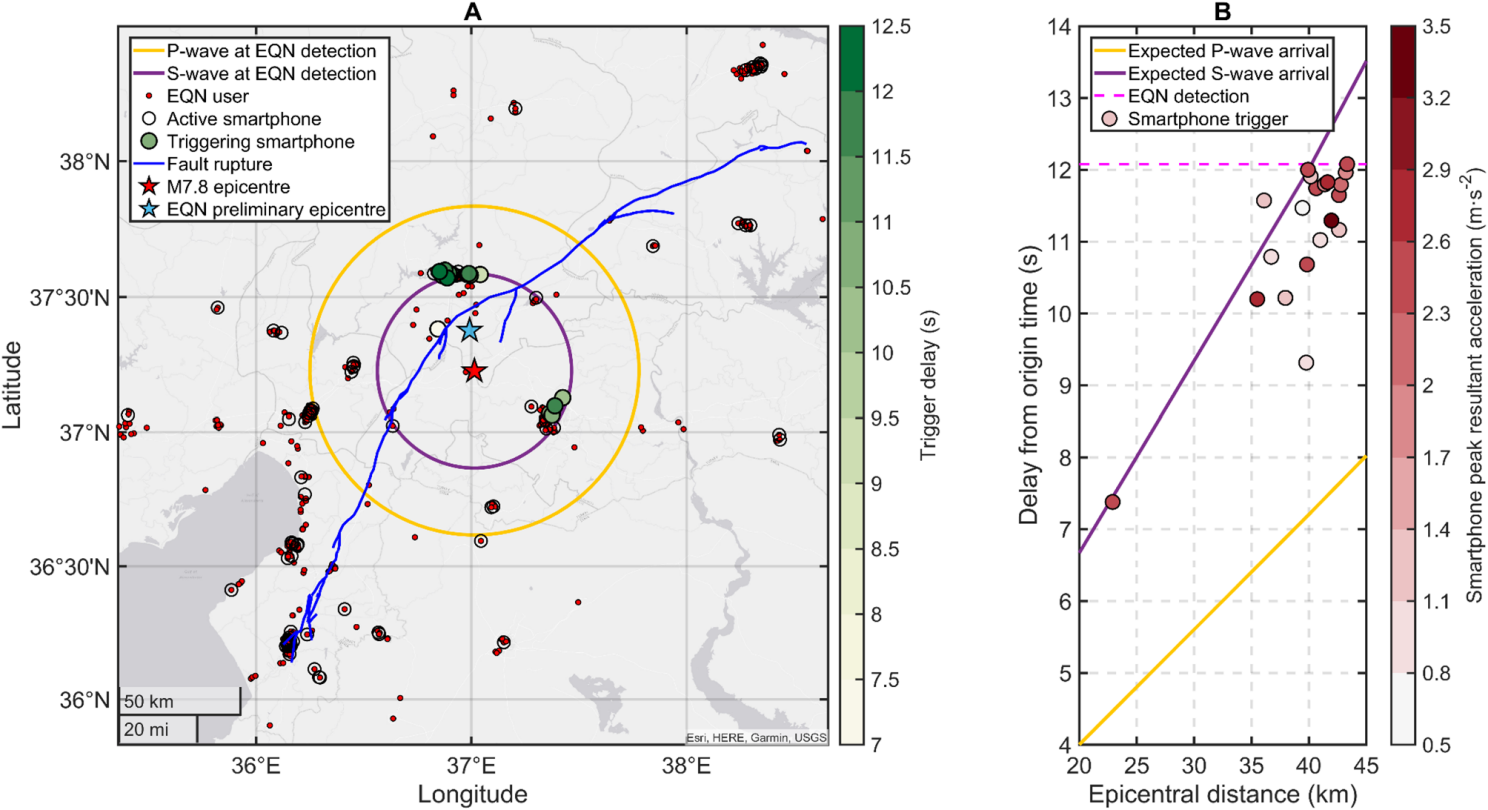
Relevant information regarding the EQN detection of the M7.8 Pazarcik earthquake on 6 February 2023. (**A**) State of the EQN smartphone network at the time of the earthquake detection, including: smartphones that detected the earthquake (filled circles), active monitoring smartphones (transparent circles) and smartphones with the EQN app installed but not monitoring (red circles). Map generated by authors using the MATLAB R2023a software. (**B**) Smartphone triggering delay (s) from origin time versus epicentral distance (km).

Figure 1 details the smartphone network configuration at the time of the EQN detection. The figure highlights the smartphones that detected the earthquake (triggering smartphones), the smartphones that were monitoring at the time of the EQN detection (but not yet triggered) and the smartphones with the EQN app installed (but not charging and thus not monitoring). The estimated locations of the P- and S-waves at the time of EQN detection reveal that the smartphones triggered on the P-wave.

### Alerting strategy

The alert was distributed to EQN users located within circular areas centred on the estimated epicentre, with a radius determined from the intensity predictive equation described in the Methods section. At t0 + 12.78 s and after 0.68 s of the EQN detection time—a delay necessary to assess the shaking level and to identify the EQN users requiring an alert—the EQN issued the alert with a warning for strong ground shaking to 382 EQN users (active or not) within a radius of 141 km, with a warning for moderate shaking to 738 users within a radius of 141–412 km (in Türkiye but also Syria, Cyprus and Lebanon), and with a warning for mild shaking to 62,419 users (in Türkiye and many other countries) within a radius of 412–3506 km. The ability to transcend boundaries is a key feature that differentiates EQN from national warning systems.

### Warning time analysis

The effectiveness of the EQN alerting service is assessed by calculating the spatial distribution of the warning time and the warning time given to smartphone users with the EQN app installed at the time of the magnitude 7.8 event.

We evaluate the warning time as the time difference between the EQN alert (given at t0 + 12.78 s) and when the peak ground acceleration (PGA) measured on accelerometric stations exceeds 12%g, with g being the Earth’s acceleration of gravity. The 12%g PGA threshold corresponds to the modified Mercalli intensity of VI^20^. Thus, the warning time is calculated for a level of ground shaking at which people can still take protective measures.

Waveforms collected by the stations of the Turkish Disaster and Emergency Management Authority (AFAD) were initially analysed to determine the geographical region where the PGA surpassed 12%g. Within this area, the warning time was estimated at the AFAD stations and interpolated over space using statistical modelling (see “Methods” section).

**Figure 2:**
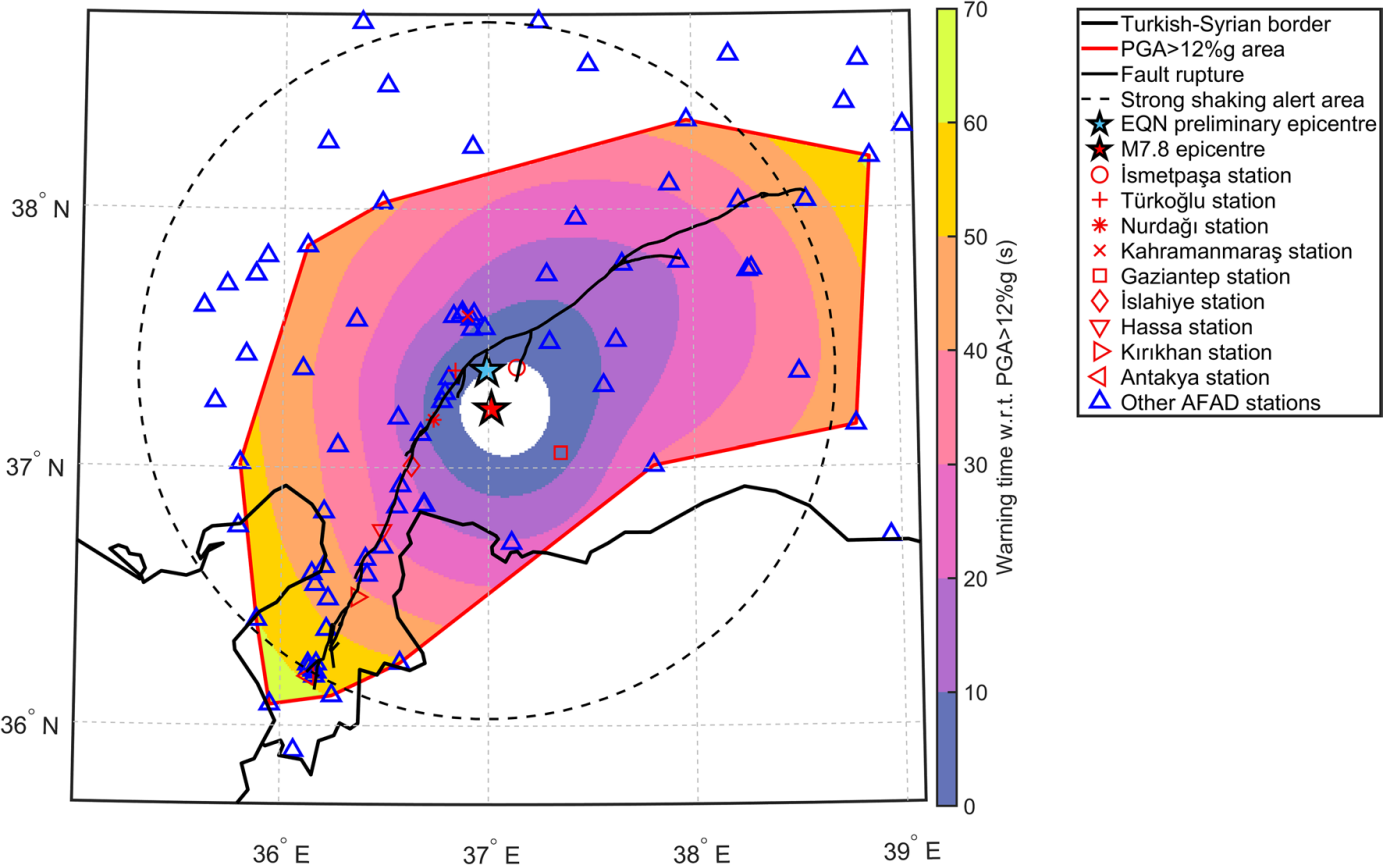
Spatial distribution of the warning time (s), with respect to the exceedance of the 12%g PGA threshold, provided by the EQN system during the M7.8 Pazarcik earthquake. Map generated by authors using the MATLAB R2023a software.

Figure 2 reveals that the warning time extended up to 66 s within the 12%g exceedance region and up to 58 s within the 141 km radius of strong ground-shaking warning. Table 1 details the minimum and maximum warning times for some ranges of epicentral distance. Due to the anisotropy of the warning time, its variability in each range is relatively high.

**Table 1:** Minimum and maximum warning time for EQN users within a given epicentral distance range.

		Epicentral distance range (km)					
		[0, 25]	(25, 50]	(50, 75]	(75, 100]	(100, 125]	(125, 150]
Warning time (s)	Minimum	0.0	0.0	8.0	15.1	20.5	29.5
	Maximum	6.4	25.6	39.4	49.8	56.8	63.8

**Figure 3:**
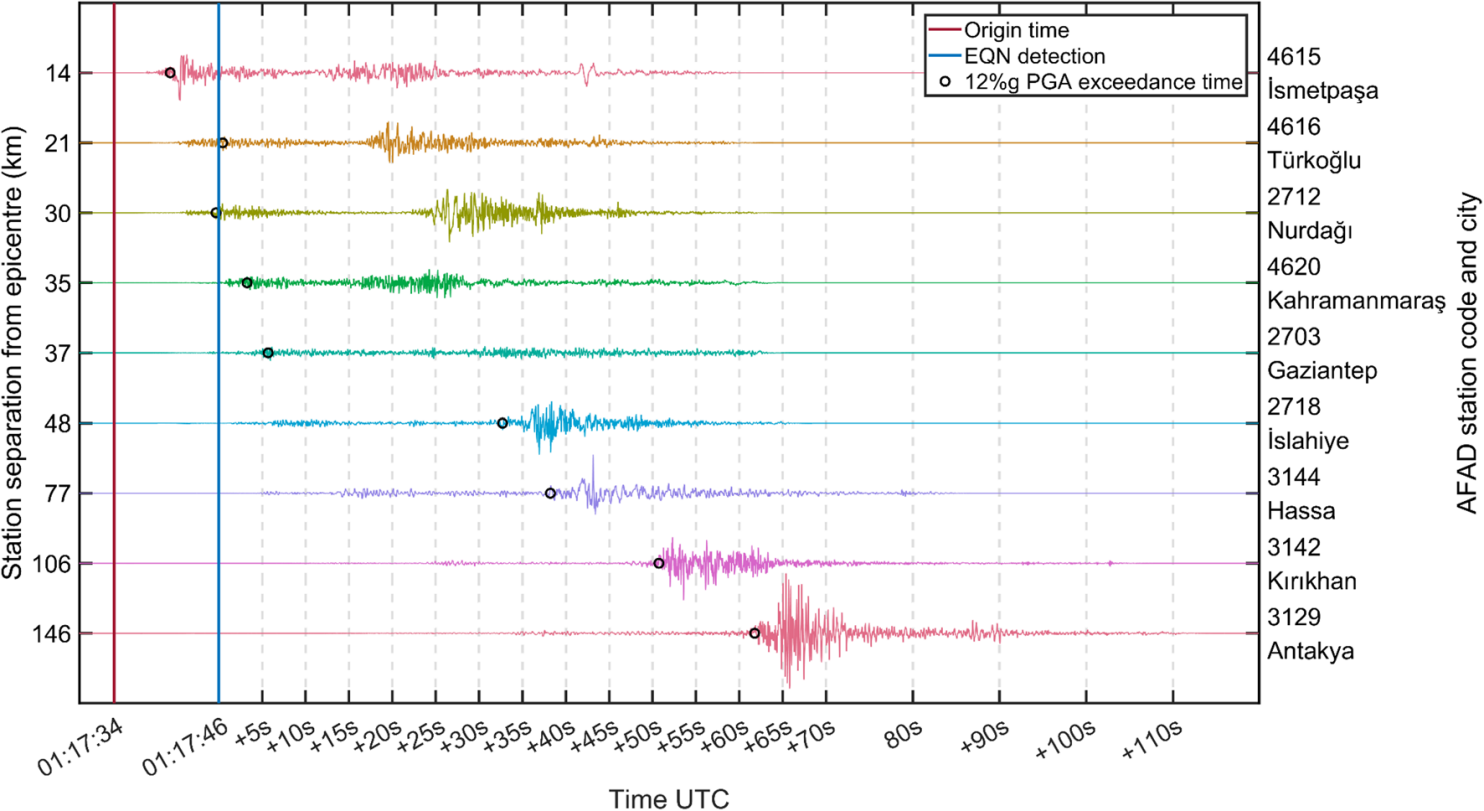
Waveforms of the east–west component recorded by AFAD stations located in major cities and towns affected by the M7.8 earthquake of 6 February 2023. The warning time at the station is the difference between the EQN alert time and the time when PGA exceeded 12%g.

**Figure 4:**
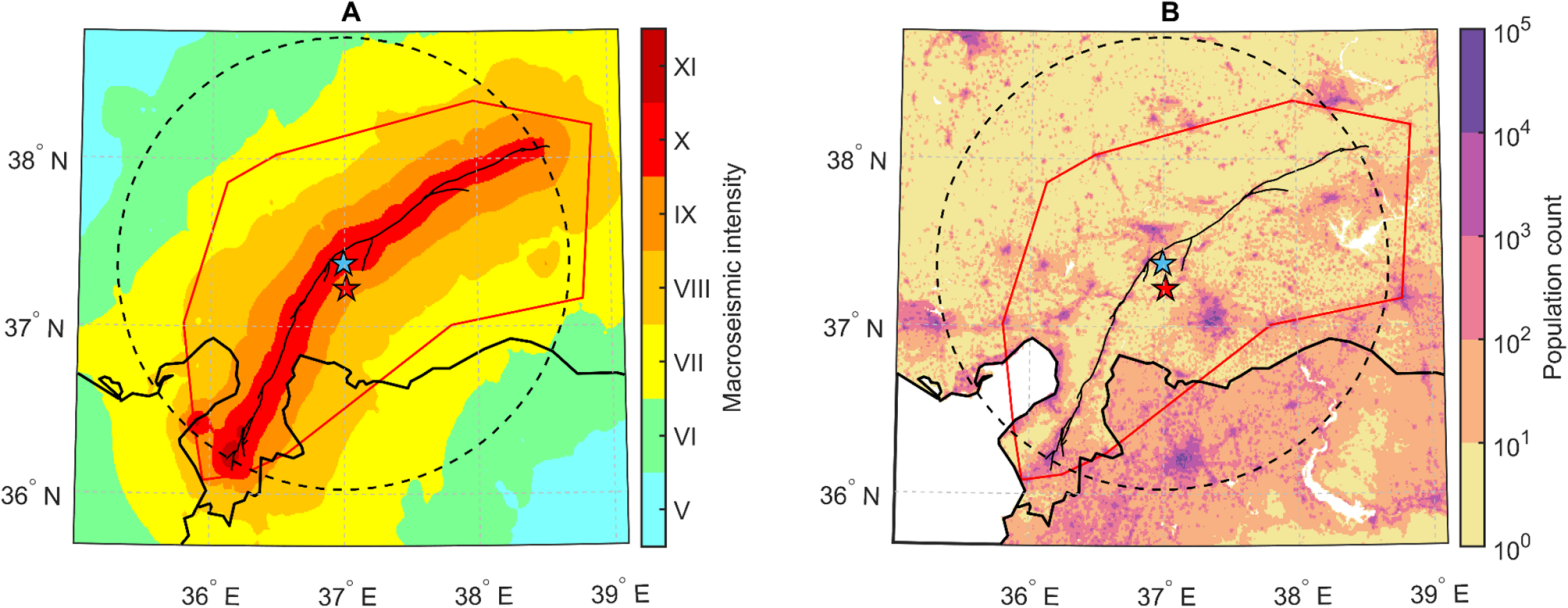
(**A**) Spatial distribution of macroseismic intensity^23^. (**B**) Spatial distribution of the population. The Fault rupture is taken from Reitman et al. (2023), while macroseismic intensity is taken from Hancilar et al.^23^. Legend in Fig. 2. Maps generated by authors using the MATLAB R2023a software.

Figure 3 illustrates, for a few selected AFAD stations located in major cities and towns, the waveform plots of the east–west component (the largest component due to the effects of fault-normal directivity) recorded by the station accelerometer. A few cities and towns that experienced high levels of ground shaking benefitted from relatively long warning times. This is a consequence of the rapid EQN detection and the length of the fault rupture. The AFAD station in Antakya, 146 km from the epicentre (see Fig. 2), recorded a PGA of 138%g and the macroseismic intensity within the city reached an intensity of XI on the modified Mercalli scale (see Fig. 4). EQN users living in Antakya received an alert approximately 60 s before the 12%g PGA was exceeded, but the warning was for moderate ground shaking rather than strong ground shaking. This is because the city is approximately 160 km from the epicentre estimated by the EQN, thereby exceeding the 141 km radius of the strong-shaking warning. Table 2 shows how many EQN users received a warning for moderate shaking even though they were exposed to strong shaking (i.e. macroseismic intensity equal to or greater than VI). However, the warning time for users outside the 141 km radius was more than 40 s (see Fig. 2). This means that, despite the underestimated shaking level, all users had enough time to respond to the alert.

**Table 2:** Number of EQN users who received a warning for strong and for moderate shaking, classified by their macroseismic intensity.

		Macroseismic intensity								Total
		< V	V	VI	VII	VIII	IX	X	XI	
Numbers of EQN users per warning type	Strong				28	124	177	53		382
	Moderate	492	35	2	71	58	6	6	68	738

**Figure 5:**
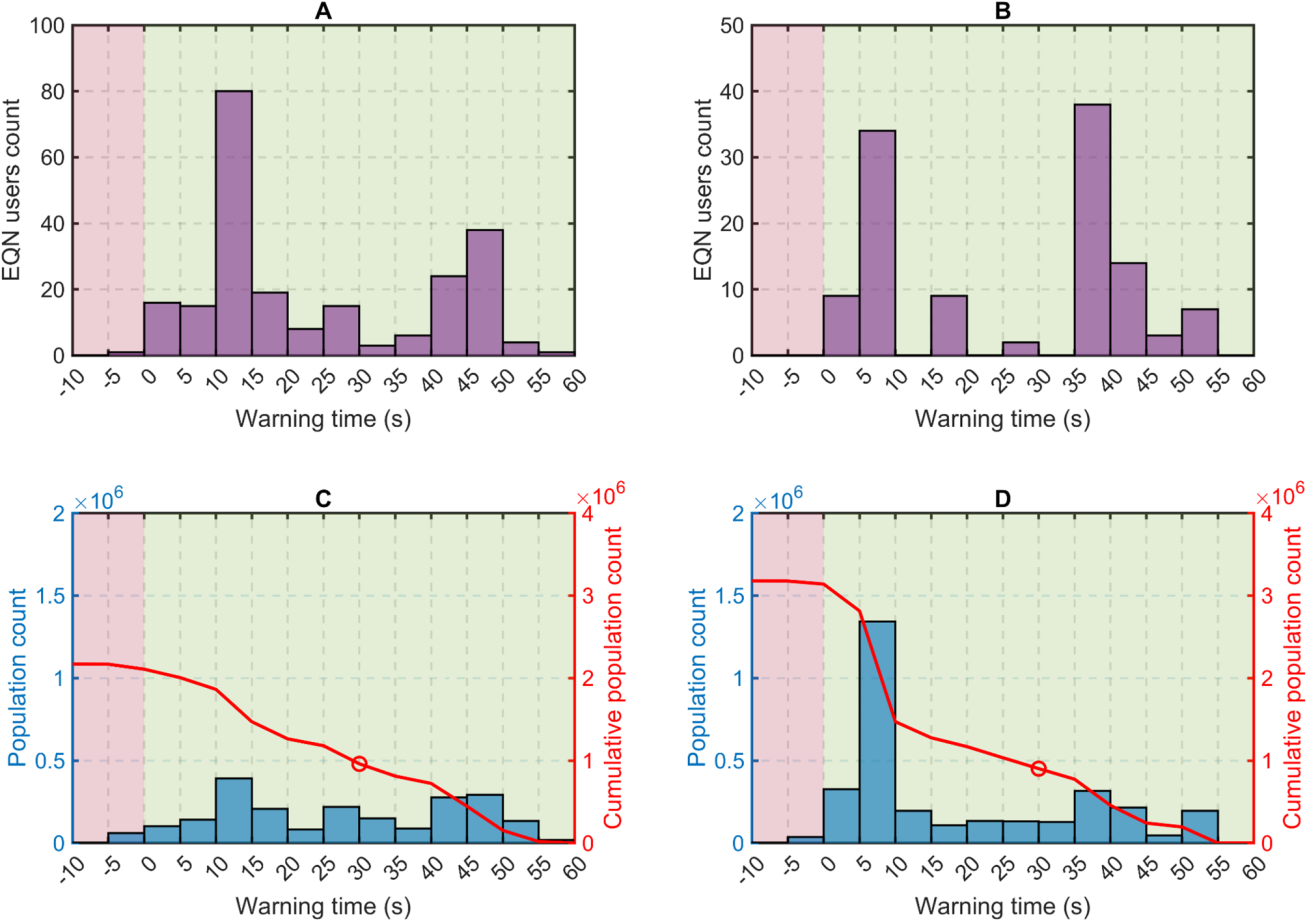
(**A**) Warning time distribution for EQN users exposed to intensity IX, X and XI and (**B**) exposed to intensity VIII. (**C**) Potential warning time distribution for the Turkish–Syrian population exposed to intensity IX, X and XI and (**D**) exposed to intensity VIII if the strong ground-shaking alert were provided to the entire population. The warning time is based on exceeding the 12%g peak ground acceleration threshold and is calculated for the EQN users and for the population located within the 141 km radius centred on the EQN preliminary epicentre. The red graph displays the number of people who could potentially have benefitted from an increased warning time than any given value. The circles in (**C**) and (**D**) represent the number of people who could potentially have benefitted from a warning time exceeding 30 s.

The estimated fatality rate in Türkiye is 1/38 for people exposed to macroseismic intensity IX and above, and 1/1000 for people exposed to intensity VIII^21^. It is interesting to estimate the actual warning time distribution for the EQN users located within the 141 km radius who received a strong ground-shaking warning. Figure 5 depicts that 230 users were exposed to macroseismic intensity of IX and above, while 124 users were exposed to intensity VIII. The maximum warning time was 58 and approximately 53 s, respectively.

### Potential warning time distribution among the Turkish-Syrian population

The warning time analysis conducted with EQN users was also applied to the Turkish–Syrian population to evaluate the potential warning time for those exposed to intensities of VIII or higher. This was done under the assumption that the distribution of the strong-shaking warning would have encompassed the entire population located within the 141 km radius (Fig. 4). While this assumption of a larger number of potential users implies a significant but manageable change in the EQN alert mechanism (see “Discussion” in the next section), it is nevertheless useful to assess the potential of the EQN as a PEEWS open to all citizens^22^.

Figure 5 indicates that approximately 2.2 million people within the radius were exposed to macroseismic intensities of IX and above and 3.2 million people to intensity VIII. A warning time of between 30 and 58 s would have been given to 960,000 and 902,000 people, respectively. On the other hand, 826,000 people exposed to intensities of VIII or higher would have received a moderate-shaking warning (thus with the intensity underestimated) with a warning time of between 38 and 66 s.

### Could EQN have warned millions of people?

A critical aspect of EQN is the ability to alert potentially millions of users. EQN relies on the Firebase Cloud Messaging (FCM) solution^24^, which enables back-end server infrastructures to send notifications and alerts to smartphone apps. The current EQN alerting rate is approximately 100,000 users per second, and the alert is distributed by ordering the users with respect to their distance to the preliminary epicentre. This rate allows EQN to provide a reliable EEW service to its users, which are currently around 2 million distributed worldwide. If EQN needed to alert millions of users in a relatively small area, the current alerting rate would not be high enough (if compared to the speed of seismic wave propagation) and EQN would have to switch to a different alerting method which is also made available by the FCM. This other method, which is likely the same one used by Google's Android Earthquake Alerts System to alert its large user base, allows to alert million of smartphones but it does not allow to control the order in which the alert is distributed and, according to the technical documentation, may have a higher latency.

Another relevant aspect is the ability for the EQN to serve the users who install and interact with the app after a felt earthquake. The EQN server infrastructure is designed to serve a certain maximum number of app users. This exposes EQN to potential service degradation (including the real-time monitoring) if the number of users increases rapidly following a widely felt earthquake. For example, after the M7.8 event, the number of Turkish users increased from 33,000 to 225,000 within a few hours and the saturation of the EQN infrastructure prevented the detection of the M7.5 Elbistan earthquake which occurred 9 h after the Pazarcik earthquake. The problem was resolved within two days by adding more servers to the infrastructure. This type of problem can be avoided by using autoscaling solutions in the cloud, but these are usually more expensive. The EQN citizen science initiative is entirely funded by citizens who voluntarily purchase the PRO version of the app or its priority services. These services allow them to be among the first 100,000 users to receive the alert, regardless of their distance from the epicentre. While this alerting scheme may slightly alter the order in which the alert is distributed, it does not prevent non-purchasing users from receiving the alert.

## Discussion

This study demonstrated the performance of the EQN smartphone-based PEEWS deployed for the first time for a large-magnitude destructive earthquake with the epicentre located in a densely populated area. Our analysis revealed that EQN users who faced life-threatening ground shaking levels received a strong ground-shaking warning with warning times of up to 58 s, and that approximately 2.7 million Turkish and Syrian citizens exposed to the same ground-shaking levels could have benefitted from strong-shaking and moderate-shaking warnings with warning times ranging between 30 and 66 s, which is sufficiently long to take protective actions.

Issuing an alert message does not guarantee that reactions to the alert will be appropriate^10^. Advance preparation and the development of a risk culture are essential to improve alert response. In the case of flood warnings, the quality of alert response is correlated with long-term preparation carried out years before the event^25^. In other words, an earthquake early warning system is not only complementary but also reinforced by the application and monitoring of earthquake-resistant building codes.

**Figure 6:**
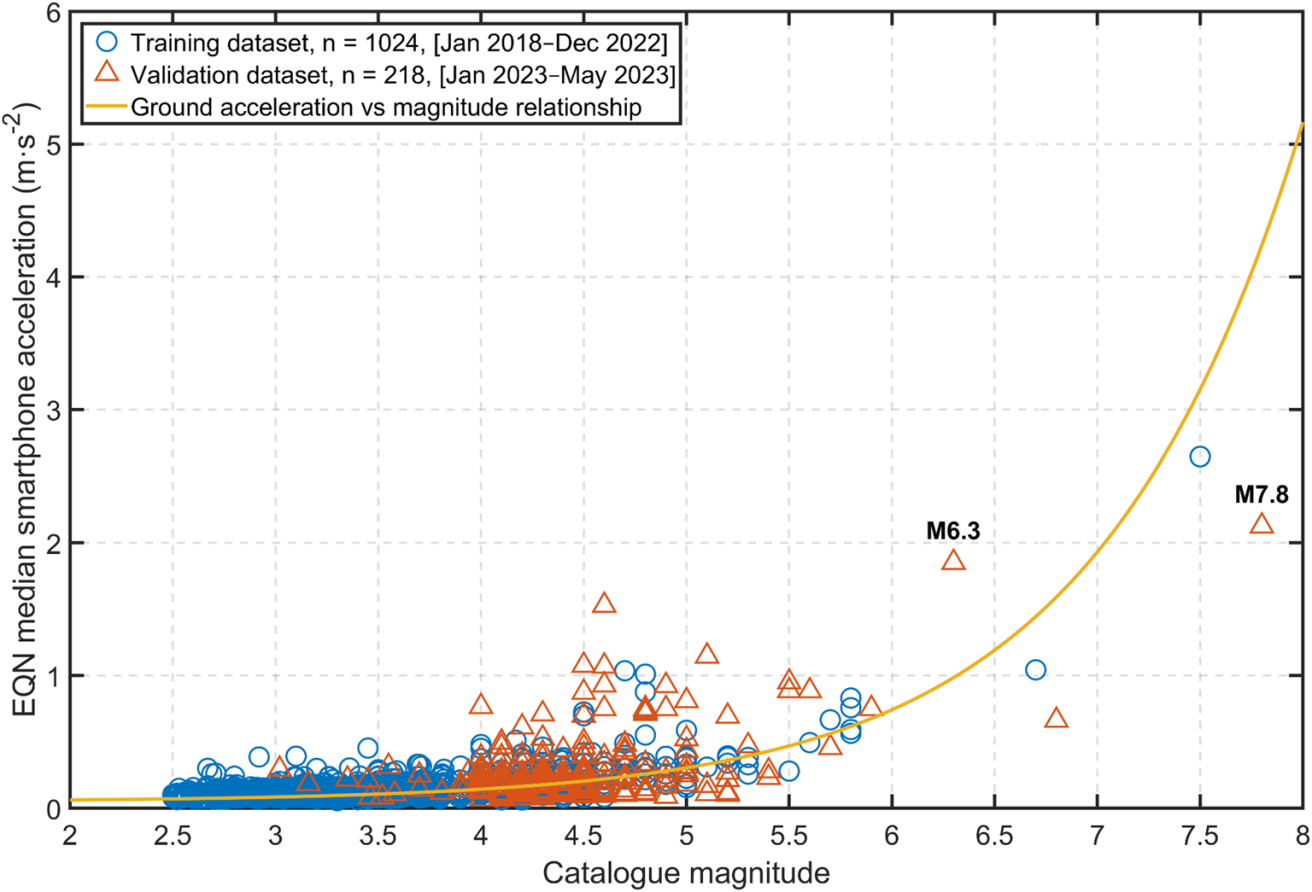
Relationship between the EQN median smartphone acceleration and the magnitude of the detected earthquake. The M7.8 event of 6 February 2023 and the M6.3 event of 20 February 2023 in Türkiye are highlighted by labels. Training and validation datasets include EQN detections with a maximum distance of 150 km between the earthquake epicentre and the EQN detection location.

The EQN alert is issued when the acceleration levels measured by the smartphones in the first few seconds of the ground shaking exceeds a given threshold. These ‘early’ vibrations are associated with the first seismic arrivals (P-wave, initial phase of seismic rupture) and the variability of the levels measured from one device to another is large due to the numerous factors affecting the vibrations measured (e.g. location in the building, soil-structure interaction, smartphone instrumental response). The acceleration levels measured by the smartphones show levels of acceleration which are different from the ones measured, on ground, by free-field seismological stations^26^. Under no circumstances can such measurements provide an accurate estimate of the size of the earthquake that is still developing when an alert is issued. However, Fig. 6 illustrates an increase in the median acceleration measured by smartphones in the first few seconds as the magnitude increases (particularly from magnitude 5.5 upwards). The corresponding empirical relationship between median smartphone accelerations and earthquake magnitude (derived using EQN data collected since 2018) underpins the EQN alert strategy. The training dataset used to establish this relationship was initially calibrated using a small number of large-magnitude data and the relationship was confirmed by the acceleration levels of the Turkish earthquake sequence. This predictability of earthquake magnitude using records of the early phase of the earthquake is consistent with seismological studies that reveal that the early phase of strong earthquakes recorded at close and regional distances is more energetic than that of weak earthquakes^27^ or with geological analysis that reveals that hypocentres are often close to regions of large slip^28^. However, this predictability has not been confirmed by a few recent studies^29^ using teleseismic records, which may indicate the important role of nearby/regional records (to which smartphone data contribute) for predictability.

Leveraging these considerations, the EQN alert strategy aims to maximise the warning time for citizens. In instances of high-magnitude events with significant fault lengths, individuals residing far from the epicentre and along the fault rupture may be vulnerable to intense ground shaking, but issuing an alert immediately after detecting an earthquake can provide them with a long warning time. The drawback of this alerting strategy is that the anticipated degree of shaking may be miscalculated for individuals situated at great distances from the epicentre at the conclusion of the fault rupture and be overestimated for those located far from the fault rupture in perpendicular directions.

Another limit of smartphone-based PEEWSs is that the network geometry mimics the spatial distribution of the population and is not designed to minimise the earthquake detection time, as done by classical PEEWSs^30^. This limitation is less critical when the risk is associated with crustal faults located in densely populated areas. In such cases, the detection delay is small and there is an opportunity to provide relatively large warning times to people exposed to high macroseismic intensities.

In the absence of a national PEEWS, a smartphone-based system such as the EQN is widely accepted by the population, as evident from the increase in the number of active users. Beginning with 33,000 users in Türkiye at the time of the M7.8 earthquake, the number rose to 1.6 million after the M6.3 aftershock on 20 February 2023. Another notable feature of smartphone-based PEEWS is its ability to transcend national boundaries due to the global reach of smartphone networks.

## Methods

### Earthquake detection and alerting strategy

The EQN system detects earthquakes in real-time by analysing the data collected by the smartphone network and sent to the EQN server. When a smartphone is charging, the EQN app enables the reading of the smartphone’s accelerometer, which provides the smartphone acceleration along the three orthogonal directions (X–Y–Z) of a three-dimensional Cartesian reference system fixed to the smartphone object.

The EQN application continuously monitors the resultant acceleration computed from the three components. If the resultant acceleration exceeds a smartphone-specific threshold, a message is sent to the EQN server over the Internet. The message includes the smartphone’s coordinates in space and the smartphone’s peak resultant acceleration (SPRA) measured over a three-second window.

The EQN server analyses all the messages received from the smartphones in real-time. An earthquake is detected using a statistical detection algorithm^31^. The output of the algorithm is the preliminary estimate of the epicentre and the list of SPRA values.

Let $\left\{{SPRA}_{(1)},\dots ,{SPRA}_{(N)}\right\}$ be the ordered set of $SPRA$ values (expressed in m/s^2^), with $N$ being the number of smartphones that contributed to the earthquake detection. To filter out anomalous values, the median smartphone acceleration (MSA) is computed as:

$$MSA=\left\{\begin{array}{cc}S{PRA}_{\left(\frac{N+1}{2}\right)}& \text{if } N \text{ is odd}\\ \left[{SPRA}_{\left(\frac{N}{2}\right)}+{SPRA}_{\left(\frac{N}{2}+1\right)}\right]/2& \text{if } N \text{ is even}.\end{array}\right.$$

The earthquake magnitude is estimated using the following relationship:

$$M={log}_{e}\left((MSA-0.050)/0.0017\right),$$

which was learned from past EQN detections and it is, thus, specific to the EQN system (Fig. 6).

During the next 30 s after earthquake detection, the EQN system updates the $MSA$ every 3 s based on the smartphone messages received by the server in the last 10 s. If the $MSA$ increases by 20% with respect to the last estimate, the magnitude $M$ is recomputed and a new alert is issued.

The EQN system alerts its users located in the geographical area where the earthquake is expected to generate mild, moderate, or strong ground shaking. The EQN assumes that the earthquake is a point source and the spatial distribution of the macroseismic intensity is estimated using an isotropic intensity predictive equation^32^:

$$I= -2.15{log}_{10}r+1.03M+2.31,$$

where $r$ is the hypocentral distance based on the preliminary EQN epicentre. Further, the IPE is inverted to obtain the hypocentral distance for any intensity value $I$:

$${r}_{I}={10}^{\left[\left(2.31-I+1.03M\right)/2.15\right]}.$$

The epicentral distance at which the earthquake intensity is equal to $I$ is given by

$${d}_{I}=2R\left(\sqrt{\frac{\left({r}_{I}^{2}-{z}^{2}\right)}{4R\left(R-z\right)}}\right),$$

where $R$ is the Earth’s radius in km and $z$ is the depth of the earthquake (preliminarily assumed to be 10 km). The alert is sent to all EQN app users located within a circular area centred on the EQN preliminary epicentre with a radius of ${d}_{I = 2}$ km. The app customises the warning message based on the distance of the smartphone from the epicentre. The message reads “Expect intense shaking” for users within a radius of ${d}_{I = 5}$ km, “Expect moderate shaking” for users within a radius of between ${d}_{I = 5}$ and ${d}_{I = 4}$, and “Expect mild shaking” for users within a radius of between ${d}_{I = 4}$ and ${d}_{I = 2}$.

**Figure 7:**
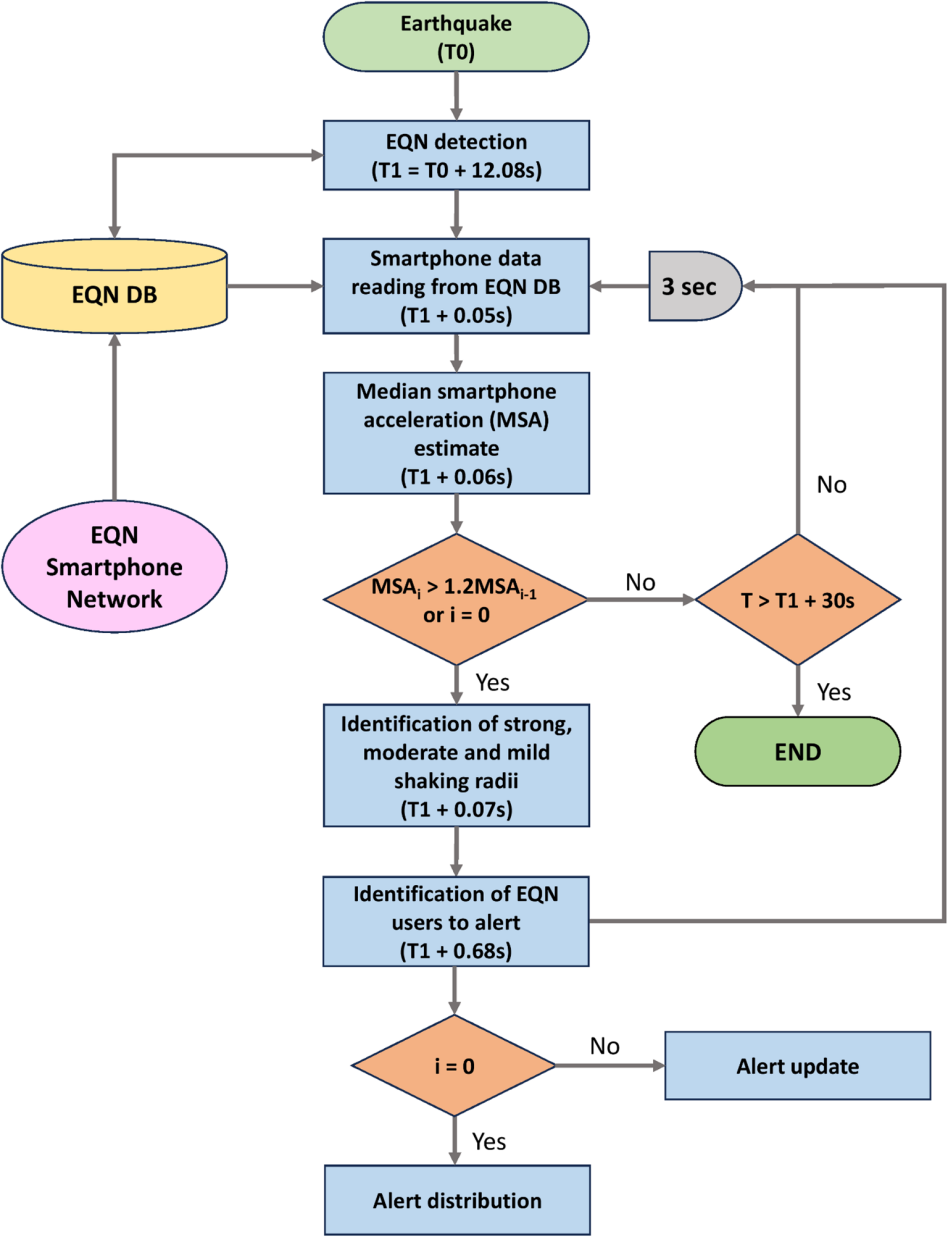
EQN alerting strategy. Times refer to the M7.8 Turkish-Syrian earthquake detected by the EQN system.

EQN issues an alert each time $M$ is updated, but the EQN application only displays the new alert to the smartphone user if the expected shaking shifts from mild to moderate or from moderate to strong after the original alert. Figure 7 illustrates the EQN alerting strategy.

### Calculation of warning time

The waveforms of the AFAD stations illustrated in Fig. 2 were analysed to estimate the spatial distribution of the warning time in relation to the exceeding of the 12%g PGA threshold. Let ${PGA}_{i,t}$ be the PGA at the i-th station up to time $t$. Then, the exceedance time at i-th station is defined as

$${t}_{i}^{*}={min}_{t}\left({PGA}_{i,t}>12\%g\right),$$

while the warning time at the station is given by ${w}_{i}={t}_{i}^{*}-{t}_{a}$, where ${t}_{a}$ is the time at which the EQN alert is issued.

Quantities ${PGA}_{i,t}$, ${t}_{i}^{*}$, and ${w}_{i}$ are only measured at the station location. To estimate the warning time across space, it is assumed that the $P{GA}_{i,t}$ values are observations of a spatio-temporal process continuous in space and time. This implies that ${w}_{i}$ represents observations from a continuous spatial process $w\left({\varvec{s}}\right)\in {\mathbb{R}},s\in {\mathcal{S}}^{2}\subset {\mathbb{R}}^{3},$ with ${\mathcal{S}}^{2}$ being the surface of the sphere embedded in ${\mathbb{R}}^{3}$.

The observed warning time ${w}_{i}$ is modelled as

$${w}_{i}\left({{\varvec{s}}}_{i}\right)={\beta }_{0}+{\beta }_{1}{d}_{i}+\alpha \omega \left({{\varvec{s}}}_{i}\right)+{\varepsilon }_{i},$$

where ${d}_{i}$ is the distance from the earthquake epicentre to the AFAD station located at ${{\varvec{s}}}_{i}$, $\omega$ is a zero-mean spatial Gaussian process, and ${\varepsilon }_{i}$ is a zero mean normally distributed random error with variance ${\sigma }_{\varepsilon }^{2}$.

The role of $\omega$ is to capture the residual spatial variability of ${w}_{i}$, which is not described by the linear relationship ${\beta }_{0}+{\beta }_{1}{d}_{i}$. The spatial correlation function of $\omega$ is $\rho \left({{\varvec{s}}}_{i},{{\varvec{s}}}_{j}\right)=exp\left(-\frac{\| {{\varvec{s}}}_{i},{{\varvec{s}}}_{j}\| }{\theta }\right)$, where $\| {{\varvec{s}}}_{i},{{\varvec{s}}}_{j}\|$ is the geodetic distance between any two points in space ${{\varvec{s}}}_{i}$ and ${{\varvec{s}}}_{j}\in {\mathcal{S}}^{2}$.

Model parameters are ${\beta }_{0},{\beta }_{1},{\sigma }_{\varepsilon }^{2},\alpha$ and $\theta$, and they are estimated using the expectation–maximization algorithm implemented by the D-STEM v2.0 software^33^ (github.com/graspa-group/d-stem). For any spatial location ${\varvec{s}}$, the estimated warning time is given by

$$\widehat{w}\left({\varvec{s}}\right)= {\widehat{\beta }}_{0}+{\widehat{\beta }}_{1}d\left({\varvec{s}}\right)+\widehat{\alpha }\widehat{\omega }\left({\varvec{s}}\right),$$

where ${\widehat{\beta }}_{0}$, ${\widehat{\beta }}_{1}$, and $\widehat{\alpha }$ are the estimated model parameters, $d\left({\varvec{s}}\right)$ is the epicentral distance, and $\widehat{\omega }\left({\varvec{s}}\right)$ is the Gaussian process $\omega$ predicted at location ${\varvec{s}}$^34^.

The warning time depicted in Fig. 2 is $\widehat{w}\left({\varvec{s}}\right)$ and is estimated within the alpha-shape polygon defined by the spatial locations of the AFAD stations at which the 12%g PGA threshold was exceeded.

### Population exposure vs warning time

Population exposure is assessed through macroseismic intensity^23^ and population distribution^35^ (Fig. 4). The intensity map grid is based on pixels with a spatial resolution of approximately 1.77 × 1.77 km. The population spatial resolution is approximately 1.00 × 1.00 km and each pixel indicates the number of people living in the area covered by the pixel. To align the two maps, the intensity map is interpolated over the grid of the population map under the assumption that the intensity values refer to the centres of the grid pixels. Finally, the warning time $\widehat{w}\left({\varvec{s}}\right)$ is estimated at the spatial locations given by the centres of the population grid pixels. In this manner, macroseismic intensities, population counts, and warning times are available on the same grid. This enables us to calculate the potential warning time distribution over the population for different macroseismic intensity ranges (Fig. 5).

## Data Availability

Data pertaining to the detection of the M7.8 Pazarcik earthquake of 6 February 2023 by the EQN have been provided by Futura Innovation SRL under the research agreement with the first author. The software for reproducing the data analysis is available at https://zenodo.org/records/10621953.
